# A Data-Driven Method for the Estimation of Truck-State Parameters and Braking Force Distribution

**DOI:** 10.3390/s22218358

**Published:** 2022-10-31

**Authors:** Qunyi Chu, Wen Sun, Yuanjian Zhang

**Affiliations:** 1 Arts and Sciences, New York University Shanghai, Shanghai 200122, China; 2 College of Automotive Engineering, Changzhou Institute of Technology, Changzhou 213028, China; 3 Aeronautical and Automotive Engineering Department, Loughborough University, Loughborough LE11 3TT, UK

**Keywords:** state estimation, data processing, feature filtering, braking force distribution strategy, generalized regression neural network (GRNN)

## Abstract

In the study of braking force distribution of trucks, the accurate estimation of the state parameters of the vehicle is very critical. However, during the braking process, the state parameters of the vehicle present a highly nonlinear relationship that is difficult to estimate accurately and that seriously affects the accuracy of the braking force distribution strategy. To solve this problem, this paper proposes a machine-learning-based state-parameter estimation method to provide a solid data base for the braking force distribution strategy of the vehicle. Firstly, the actual collected complete vehicle information is processed for data; secondly, random forest is applied for the feature screening of data to reduce the data dimensionality; subsequently, the generalized regression neural network (GRNN) model is trained offline, and the vehicle state parameters are estimated online; the estimated parameters are used to implement the four-wheel braking force distribution strategy; finally, the effectiveness of the method is verified by joint simulation using MATLAB/Simulink and TruckSim.

## 1. Introduction

Economic development has placed stricter demands on the transportation industry, and the increased use of commercial vehicle transportation as a mode of road transportation for the transport of goods and passengers has played a vital role in easing the pressure on transportation and increasing the productivity of various industries [1,2,3]. Among them, the braking of commercial vehicles is an important basis for ensuring the safety of vehicle driving and the development of active safety technology, and the emergence of electro-mechanical brake (EMB), which is responsive, easy to realize active braking, and offers more accurate control of braking force, provides a strong hardware basis for intelligent vehicles [4,5]. EMB provides executable prerequisites for the intelligent braking of the vehicle under different working conditions to ensure driving safety [6,7].

In terms of active safety functions, the anti-lock brake system (ABS), which actively intervenes in the braking force, plays an important role in improving the car’s wheel adhesion capacity and driving stability during heavy braking, and the control strategy of ABS can be mainly divided into rule-based control strategy [8,9,10] and model-based control strategy [11,12,13]. Among them, the rule-based control strategy considers the state of the vehicle driving with the slip rate as the limit and also considers the angular deceleration rate for the decision of discontinuous control such as increase, pressure preservation, and decompression. This control method is computationally small, the technology is relatively mature, and the accuracy requirement for the state observation of the vehicle is small, and owing to the high stability of this control method, this method is commonly used in various ABS strategy development. However, due to the inability to directly measure the slip rate, the accuracy of vehicle observation of slip rate, and the limitation of non-continuous control, the method can only perform suboptimal control in practice, and does not take advantage of EMB’s precise control of each wheel braking force. Therefore, the traditional rule-based control strategy is difficult to balance the control adaptability and real-time control effect.

In the research on model predictive control strategy, the application of the MPC method of ABS control is gradually becoming a research hotspot, which requires fewer calibration parameters compared with the traditional controller, thus shortening the development time, and the method can adjust the vehicle braking torque in real time by following the vehicle slip rate, which can precisely control the braking force of each wheel under the vehicle braking torque distribution strategy. However, due to the huge computational volume of the model predictive control, it is difficult to meet the requirements of real-time control due to the limitation of the computing power of the automotive computing platform, so the optimization of this method is also being carried out gradually [14,15].

The tire force is the fundamental cause of changing the vehicle motion state, the tire force is the basis for controlling the vehicle state, and there is a transparent mapping relationship between the tire force and the vehicle state [16,17]. There are three main traditional methods for tire force observation: filter-based methods [18,19], mathematical model-based methods [20,21], and sensor-based observation methods [22,23,24]. The filter-based methods are mainly Kalman filter and least squares [24,25], but this method is significantly affected by environmental factors in practical application, and the observation accuracy depends on the accuracy of the specified tire model. The mathematical model-based method is mainly the Romberg method [26]; the accuracy of tire characteristic parameters is required by this method. The sensor-based observation methods are very expensive, easy to be affected by the motion state of the vehicle and has poor robustness.

The problem of observing the tire force can be transferred to the solution of obtaining the mapping relationship between the vehicle motion state and the running attitude and the tire force. Because of the highly nonlinear relationship between them, the highly nonlinear solver has become an important way to build a high-precision tire force observer [27,28]. Among them, machine learning has become a critical approach to solving the problem due to its powerful generalization and inference regression capabilities. Machine learning already has a wide range of applications in the field of state observation. For example, battery SOC state observation [29,30], future vehicle speed prediction [31,32], and torque observation of key power components of hybrid vehicles [33,34]. Therefore, this method has good observation accuracy in performing state observation.

In summary, this paper proposes an improved ABS control strategy based on a high-precision state observer. In the control strategy, the wheel force high-precision state observer is integrated with the ABS control strategy to map high-precision tire force to provide an accurate reference for vehicle ABS control. The independent distribution of multi-wheel braking pressure provides a guarantee to enhance the braking efficiency of the vehicle. Finally, the joint simulation verifies wheel force state observation and vehicle ABS control accuracy.

Innovation point:

1. For the component parameters of four-wheeled trucks, which are difficult to observe accurately due to their complex nonlinear variations, this paper proposes a data-driven machine learning estimation method to accurately estimate the vehicle parameters (this parameter specifically refers to the four-wheeled vertical force)
2. According to the estimated four-wheel force, the braking force is distributed according to the proportion of the force.
3. By collecting the operating data of the vehicle under Changchun City working conditions, the data were interpolated and filtered to eliminate component signal frequency inconsistencies, noise, and anomalies, and to construct a machine learning model dataset.

## 2. Model and Parts Introduction

### 2.1. The Model Construction of Truck

**Figure 1:**
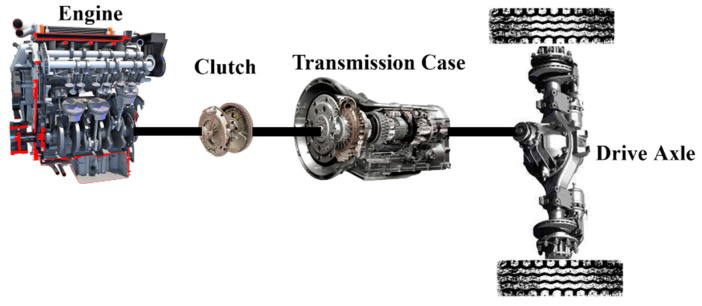
The schematic of the truck configuration.

The main research object of this paper is a four-wheel truck whose structure is shown in Figure 1. This vehicle consists of the engine, clutch, transmission, drive axle, and other components. The engine is the only power source to provide the vehicle driving energy. The vehicle is equipped with a six-speed gearbox to realize everyday driving under multiple working conditions according to information such as vehicle speed and power while improving the engine’s working point and optimizing the engine’s efficiency to a certain extent. Table 1 shows the information on the vehicle parameters of the four-wheeled truck.

### 2.2. Mathematic Model of Truck

In the actual drive-over driving process of the vehicle, to realize the normal driving of the vehicle, the power provided by the vehicle must overcome the rolling resistance, air resistance, slope resistance, and acceleration resistance, the driving force, and power balance equation expression as shown in Equations (1) and (2):

(1) $$F_{t}=mgf\cos\alpha_{slope}+\frac{C_{D}Av^{2}}{21.15}+mg\sin\alpha_{slope}+\delta m\frac{d}{dt}v$$

(2) $$P_{req}=\frac{v}{\eta_{t}}(\frac{mgf\cos\alpha_{slope}}{3600}+\frac{C_{D}Av^{2}}{76140}+\frac{mg\sin\alpha_{slope}}{3600}+\frac{\delta ma}{3600})$$

where $F_{t}$ is the tangential driving force generated by the driving wheels; m is the vehicle mass; g is gravitational acceleration; f is rolling resistance coefficient; $\alpha_{slope}$ is the road slope; $C_{D}$ is the air resistance coefficient; A is the windward area; δ is the rotating mass conversion factor; v is the vehicle speed; $T_{req}$ is the demand torque; r is the wheel rolling radius, the relevant parameter values are shown in Table 2; $\eta_{t}$ is the transfer efficiency. 

### 2.3. Engine Model

**Figure 2:**
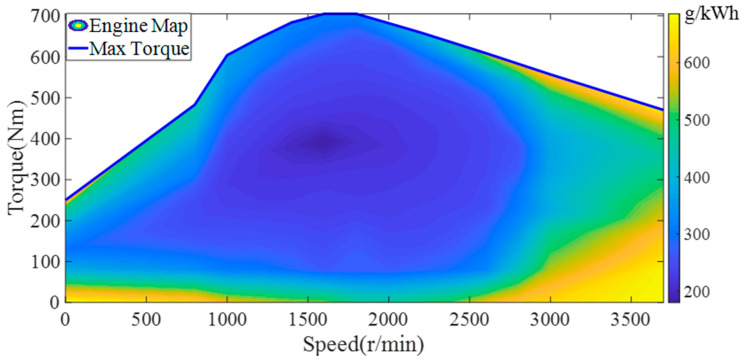
The fuel consumption map of the engine.

As the only power source of the vehicle, the maximum torque of the engine can reach 740.6 Nm. According to the experimental data, the external characteristics and efficiency map of the engine are established, as shown in Figure 2.

### 2.4. Pneumatic ABS Solenoid Valve Model

**Figure 3:**
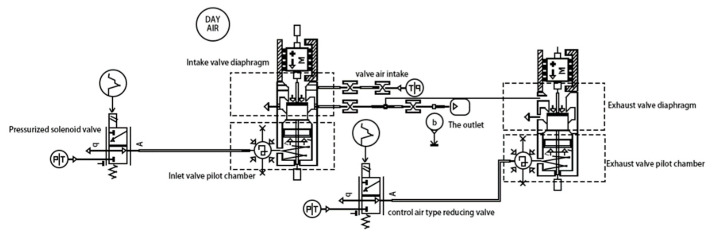
The structure diagram of ABS solenoid valve. (T is the torque; P is the pressure; A is the trachea; b is the outlet; left M is the intake cavity; right M is the exhaust cavity).

The EBS system in this paper uses dual-channel control for the rear axle and a single-channel control and ABS solenoid junction for the front axle. The ABS solenoid valve is used to control the braking pressure of the left and right wheels of the front axle separately, the braking pressure of all four wheels of the EBS system can be controlled independently. Additionally, the ABS solenoid valve consists of a booster valve and a pressure-reducing valve, of which the booster valve is a normally open solenoid valve. The structure diagram of ABS solenoid valve is shown in Figure 3 and the operating characteristics of ABS solenoid valve are shown in Table 3.

### 2.5. Brake Model

High-pressure gas from the storage cylinder through the EBS system actuators finally reaches the brake chamber to produce braking pressure, so the EBS can control the braking pressure of the brake chamber. While in ABS and regenerative braking control strategy, the final given is the braking force or braking torque of the wheels, which requires key conversion components of brakes. Brakes are mainly divided into disc brakes and drum brakes.

Disc brakes use a disc-shaped brake disc as the rotating element in the frictional vice. Disc brakes are small in size, light in mass, have good resistance to heat recession, and are commonly used in passenger cars. Drum brakes use a brake drum as the rotating element in the frictional vice. The braking torque generated by the brake is mainly related to the brake chamber pressure, brake contact area, braking efficiency, brake friction coefficient, effective braking radius, and brake coefficient, so the braking torque generated by the brake is shown in the following Equation (3):

(3) $$T_{b}=2P_{b}A_{b}\eta_{b}\mu_{b}r_{b}c_{b}$$

where $P_{b}$ is braking pressure; $A_{b}$ braking contact area; $\eta_{b}$ braking efficiency; $\mu_{b}$ braking friction coefficient; $r_{b}$ effective braking radius; $c_{b}$ is brake coefficient.

When $K_{b}=2A_{b}\eta_{b}\mu_{b}r_{b}c_{b}$, Equation (3) can be simplified as Equation (4).

(4) $$T_{b}=K_{b}P_{b}$$

where $K_{b}$ is the brake conversion factor, Table 4 gives the brake related parameter settings in the simulation, the units are all international system units, and we can calculate the brake conversion factor $K_{b}=0.0022$.

### 2.6. Tire Model

Tires are the only vehicle part that contacts the ground and transmits forces and moments. The longitudinal force, lateral force, and return torque to the vehicle are all generated by the contact between the tires and the ground, so the accuracy of the tire model is critical to the overall vehicle model. Nowadays, the mainstream tire models are divided into three categories: theoretical models, semi-empirical models, and empirical models. In order to improve the applicability of the model, the theoretical model, the GIM tire model, is chosen [35,36]. The GIM tire model has a high accuracy for the calculation of lateral and longitudinal forces and the model parameters are easily measured to meet the requirements of state estimation in this paper.

a. Slip rate calculation

The slip rate indicates the sliding ratio’s share in the overall vehicle form process. During the braking process, as the braking intensity increases, the rolling component of the wheel becomes less and less, while the sliding component becomes more and more. The formula for calculating the longitudinal slip rate is shown in Equation (5).

(5) $$S=\left\{ \begin{array}{l} \frac{r_{r0}\omega_{w}-u_{w}}{u_{w}}\times 100\%\begin{array}{cc} & Drive \end{array}\\ \frac{u_{w}-r_{r0}\omega_{w}}{u_{w}}\times 100\%\begin{array}{cc} & Brake \end{array} \end{array}\right.$$

b. Dynamic load calculation model

The slip rate indicates the sliding component’s share in the overall vehicle form process. During the braking process, as the braking intensity increases, the rolling component of the wheel becomes less and less. In contrast, the load of the four wheels remains unchanged during everyday driving. However, when the vehicle is braking, longitudinal axle load transfer occurs, at which time the vertical load (normal force) of each wheel is calculated as shown in the Equations (6)–(9).

(6) $$F_{zfl}=\frac{M}{L}\left[\frac{1}{2}gb+\frac{1}{2}a_{x}h_{g}+\frac{a_{y}h_{g}b}{d_{f}}\right]$$

(7) $$F_{zfr}=\frac{M}{L}\left[\frac{1}{2}gb+\frac{1}{2}a_{x}h_{g}-\frac{a_{y}h_{g}b}{d_{f}}\right]$$

(8) $$F_{zrl}=\frac{M}{L}\left[\frac{1}{2}ga-\frac{1}{2}a_{x}h_{g}+\frac{a_{y}h_{g}a}{d_{r}}\right]$$

(9) $$F_{zrl}=\frac{M}{L}\left[\frac{1}{2}ga-\frac{1}{2}a_{x}h_{g}-\frac{a_{y}h_{g}a}{d_{r}}\right]$$

where $F_{zfl}$ is front left wheel vertical load; $F_{zfr}$ is front right wheel vertical load; $F_{zrl}$ is rear left wheel vertical load; $F_{zrr}$ is rear right wheel vertical load; M is the mass; L is the axle distance; a is the distance from center of mass to front axle; b is the distance from center of mass to rear axle; $h_{g}$ is the height of car center of mass; $a_{x}$ is the longitudinal acceleration; $a_{y}$ is the lateral acceleration; $d_{f}$ is the front wheelbase; $d_{r}$ is the rear wheelbase.

## 3. Methodology

**Figure 4:**
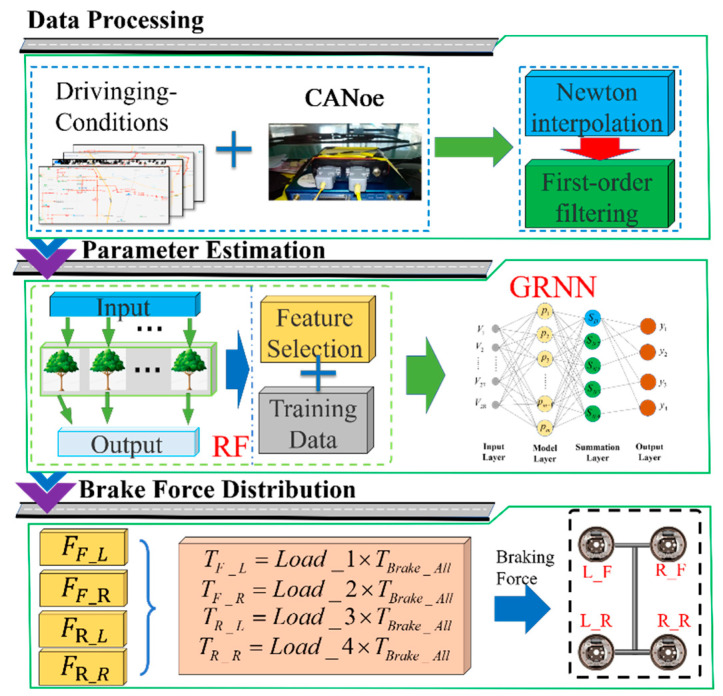
Parameter state observation and braking force distribution framework diagram. (L_F,R_F,L_R and R_R are left-front wheel brake, right-front wheel brake, left-rear wheel brake and right-rear wheel brake, respectively).

For the complex braking force system of four-wheeled trucks and the state of the vehicle and the working state of components present complex nonlinear operating characteristics, this paper proposes a machine-learning-based regression of the vehicle parameters to achieve the accurate estimation of the complex nonlinear parameters of the vehicle and improve the accuracy of the vehicle in the braking decision to give full play to the potential of the independent braking force. The architecture diagram is shown in Figure 4. Firstly, the CANoe adopted to collect information in the actual driving process of the vehicle to establish the data module. Secondly, a large number of data sets for the subsequent machine learning model training brings a huge amount of computation, the use of random forest (RF) method for the data set feature screening to reduce the dimensionality of the data and reduce the computational load. Finally, the estimation of complex nonlinear parameters of the vehicle are achieved by using generalized regression neural network (GRNN). Through the data-driven approach, the accurate estimation of the vehicle state parameters of the truck is realized, and the potential of the independent braking of the truck is fully utilized to improve the stability and safety.

### 3.1. Data Processing

In the actual driving process of the vehicle, the collected data need to be processed because of factors such as signal frequency disparity, signal noise, and abnormal data points in the collected information of the vehicle components. In this paper, Newton’s interpolation method is adopted to achieve uniformity in the time-to-time dimension of the collected data for specific differences in the signal frequencies of the collected components. In addition, the first order low-pass filtering method is adopted to process the noise and anomalies of the signals to establish an effective data set.

a. Newton interpolation

The inheritance of the Newton interpolation method can use the results of previous operations to reduce the number of operations when adding additional interpolation points. It has a more significant advantage in the process of interpolation calculation for a large amount of data. Let the time point in the original sampling frequency be less than the nearest point of the interpolated time point, marked as *β*. The data value obtained by interpolation is the average of the β−(α/2−1) data to β+α/2 data in the original sampling value.

The $x_{0},x_{1},\ldots ,x_{n}$ are n+1 non-coincident points, then the following linearly independent Newton polynomial is shown in Equation (10).

(10) $$1,x-x_{0},(x-x_{0})(x-x_{1}),\ldots ,(x-x_{0})(x-x_{1})\cdots (x-x_{n}-1)$$

Given the function value of $f(x_{i})$ exist a unique polynomial satisfying $p_{n}\left(x_{i}\right)=f\left(x_{i}\right)$, as shown in Equation (11).

(11) $$p_{n}\left(x\right)=a_{0}+a_{1}(x-x_{0})+\ldots +a_{n}(x-x_{0})(x-x_{1})\cdots (x-x_{n}-1)$$

b. First order low-pass filter

The low-pass filter achieves the characteristic is ‘pass low frequency and block high frequency’, allowing signals below the cutoff frequency to pass while signals above the cutoff frequency are blocked. This paper uses the first order low-pass filter to process the signal, and its transfer function is shown in Equation (12).

(12) $$L(s)=\frac{1}{Ts+1}=\frac{2\pi f_{c}}{2\pi f_{c}+s}$$

where T is the time constant; $f_{c}$ is the cutoff frequency, when the input signal is certain, the smaller the cutoff frequency fc is, the less signal the low-pass filter allows to pass, and the better the suppression of fluctuation. The larger the fc is, the more signal the low-pass filter allows to pass, and the greater the fluctuation. If you use a filter with a fixed cutoff frequency, you need to set the cutoff frequency fc of the low-pass filter in advance, and if it is set too high, the low-pass filter cannot play the role of filtering well.

In addition, the first order low-pass digital filtering formula is shown in Equation (13).

(13) $$Y_{n}=f(x)X_{n}+g(x)Y_{n-1}$$

where f(x) is the filter coefficient function one; g(x) is the filter function two, the actual value of the functions depends on the filter time constant and the sampling period; $X_{n}$ is the input value at the *n*-th sampling; $Y_{n}$ is the output value at the nth sampling, and $Y_{n-1}$ is the output value at the previous sampling. The first order low-pass filtering method uses the weighting of the current sampling value with the last filtered output value to obtain a practical filtered value, which makes the output have a feedback effect on the input.

The filtering effect is mainly influenced by the filter function f(x) and the filter function g(x), and there is a certain relationship between f(x) and g(x). When the actual value of f(x) is larger at a certain moment, the actual value of g(x) will decrease accordingly, and the smaller f(x) is, the smoother the filtering effect will be, and the larger f(x) is, the more unstable the filtering effect will be, with great sensitivity.

The data processing through data interpolation and filtering solves the inconsistency of data in the time dimension, eliminates the noise and anomalies of the signal, makes the data more rational and more convincing, and provides a solid basis for the establishment of data sets as well as data applications.

### 3.2. RF-Based Feature Screening

Random forest is integrated learning based on decision trees, which essentially addresses the inherent shortcomings of individual models as integrated learning algorithms and integrates individual regression models to form better models. On the basis of a large amount of data, random forest can play a better feature screening property. The feature selection in a random forest is similar to the random selection of datasets, where the decision tree in a random forest randomly selects certain features from all the features to be selected and does not use all the data features in the data. In the process of random forest feature selection, the features are selected based on the principle of the magnitude of the contribution of each feature to achieve dimensionality reduction of the data.

In the process of model training, RF adopts a bagging framework to generate m training sets by the bootstrap method. Each decision tree has inconsistencies in training samples, and the characteristics of the samples are extracted from various aspects. The sampling formula is shown in Equation (14). Input samples α have a total of n data samples in the bootstrap, and a training sample β is obtained from random sampling n times of replacement in the N samples, so a number of samples have not been collected. In order to reduce the phenomenon of over-fitting, an “out-of-bag estimate” is performed on the generalization error of the decision tree by using the original samples that are not selected. The out-of-bag estimate of samples is shown in Equation (16).

(14) $$\alpha_{N\times D}\geq \beta_{n\times d}$$

(15) $$\begin{array}{cc} s.t. & N>n\\ & D>d \end{array}$$

(16) $$H^{oob}(x)=\arg\underset{y\in D}{\max}\sum_{t}^{T}II(h_{t}(x)=d)\cdot II(x\notin N)$$

where α are the input samples; β are the training samples; N is the number of input samples; n is the number of training samples; D is the number of input samples features; d is the number of training samples features; $H^{oob}(x)$ is the out-of-bag estimate of sample *x*; $h_{t}$ is the *t*-th decision tree; T is the total number of decision trees; y is the featured item in the sample feature set.

Then the out-of-bag estimate of bagging generalization error is shown in Equation (17).

(17) $$e^{oob}=\frac{1}{D}\sum_{(x,y)\in N}II(H^{oob}\neq y)$$

In the actual application of RF, the result of the decision trees output adopts the mean value method to obtain the final prediction result of RF, as shown in Equation (18).

(18) $$V_{Future}=\sum_{t=1}^{T}v_{t}P_{t}$$

where $v_{t}$ is the speed information output by the *t*-th decision tree; $P_{t}$ is the probability distribution of the output speed information of the *t*-th decision tree.

The out-of-bag data error rate evaluates the importance of different features in the dataset. The dimensionality of the high-dimensional data is reduced by selecting the data with higher importance and excluding the data with lower importance.

### 3.3. GRNN-Based Parameter Estimation

Generalized Regression Neural Network (GRNN) as a radial-based neural network structure has strong nonlinear insertion ability as well as high fault tolerance and robustness and has a good performance in the nonlinear solution process. The theoretical basis of generalized regression neural networks is nonlinear regression analysis, where the regression analysis of the non-independent variable *Y* with respect to the independent variable *X* is actually the calculation of y with a maximum probability value, then the regression of *y* with respect to *X* (i.e., the conditional mean) is shown in Equation (19).

(19) $$\bar{Y}=E(\frac{y}{X})=\frac{\int_{-\infty }^{+\infty }yf(X,y)dy}{\int_{-\infty }^{+\infty }f(X,y)dy}$$

where $\bar{Y}$ is the predicted output of Y given the input X; f(X,y) is the joint probability density function of the random variable x and the random variable y; and X is the observed value.

Applying the Parzen nonparametric estimation, the density function can be estimated from the sample data set, as shown in Equation (20).

(20) $$\bar{f}\left(X,y\right)=\frac{1}{n{\left(2\pi \right)}^{\frac{P-1}{2}}\delta^{P+1}}\sum_{i=1}^{n}\exp\left[-\frac{{\left(X-X_{i}\right)}^{T}\left(X-X_{i}\right)}{2\delta^{2}}\right]\exp\left[-\frac{{\left(X-Y_{i}\right)}^{2}}{2\delta^{2}}\right]$$

where $X_{i}$, $Y_{i}$ are the sample observations of the random variables; n is the sample size; p is the dimensionality of the random variable; and δ is the width coefficient of the Gaussian function, here called the smooth factor. The output of the network is $\bar{Y}$. 

**Figure 5:**
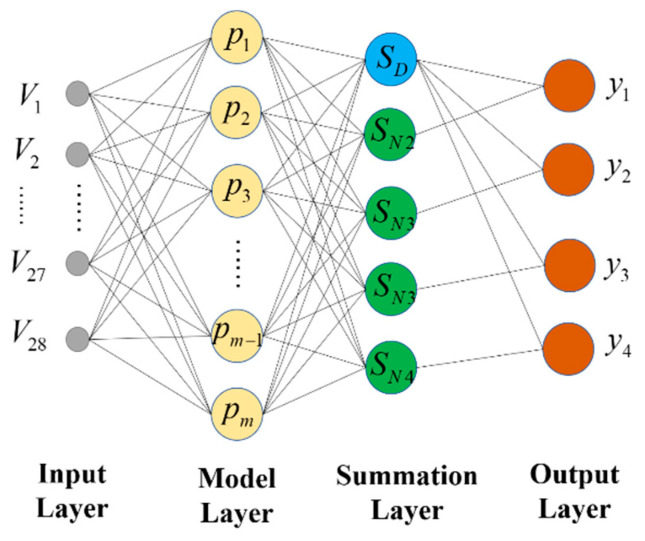
GRNN structure diagram. (V is the input; y is the output; $P_{m}$, $S_{D}$ and $S_{N}$ are node of neuron).

The GRNN network structure has four different layers: the input layer, pattern layer, summation layer and output layer, and the network structure is shown in Figure 5.

a. The input layer

The number of neurons in the input layer is equal to the dimensionality of the sample input, and the distribution of each neural unit is simple. The input variables are directly passed to the pattern layer.

b. The pattern layer

The pattern layer is fully connected to the input layer, and there is no connection between the inner layers. The number of neurons in the mode layer equals the number of input samples, and the transfer function is expressed in the exponential form of the squared Euclid distance, as shown in Equation (21).

(21) $$P_{i}=\exp[-\frac{{\left(X-X_{i}\right)}^{T}\left(X-X_{i}\right)}{2\delta^{2}}]$$

where X is the network input variable; $X_{i}$ is the learning sample corresponding to the *i*-th neuron.

c. The summation layer

The summation layer has two different ways of summing the neurons. One type of summation calculation is the arithmetic sum of the output of the mode layer with a connection weight of 1 for the mode layer and each neuron. The transfer function is shown in Equation (22). The other type of summation is calculated as a weighted sum of the neurons in the mode layer, and the transfer function is shown in Equation (23).

(22) $$S_{D}=\sum_{i=1}^{m}\exp[-\frac{{\left(V-T_{rx}{}_{i}\right)}^{T}(V-T_{rx}{}_{i})}{2\sigma^{2}}]$$

(23) $$S_{Nj}=\sum_{i=1}^{m}q_{ij}\exp[-\frac{{\left(V-T_{rx}{}_{i}\right)}^{T}(V-T_{rx}{}_{i})}{2\sigma^{2}}]\begin{array}{cc} & j=1,2,\ldots ,k \end{array}$$

where $q_{ij}$ is the weight value of the ith neuron in the model layer and the jth neuron in the summation layer;

d. The output layer

The output of the output layer node is equal to the output of the corresponding summation layer divided by the output of the first node of the summation layer. The relationship is shown in Equation (24).

(24) $$y_{k}=\frac{S_{D}}{S_{Nj}}$$

In the process of practical application, the information of the vehicle presents the characteristics of high dimensionality and multiple noise points. GRNN has high fault tolerance and robustness, and has high accuracy in the process of nonlinear parameter estimation of the vehicle.

### 3.4. Braking Force Distribution Strategy Based on Weight Coefficients

During the vehicle’s braking process, the wheels’ vertical pressure directly affects the amount of ground braking force. In the actual driving process, the reasonable distribution of braking pressure is carried out according to the estimated values of four-wheel vertical pressure (as shown in Algorithm 1).
**Algorithm 1** Online control algorithm of braking force distribution strategy.**Input:**$T_{Brake\_All}$, $F_{F\_L}$, $F_{F\_R}$, $F_{R\_L}$, $F_{R\_R}$, $S_{Brake}$ **Output:**result **Initialize:**result=[] **Notations:**$T_{Brake\_All}$ is total braking force $F_{F\_L}$ is the vertical force of the left front wheel $F_{F\_R}$ is the vertical force of the right front wheel $F_{R\_L}$ is the vertical force of the left rear wheel $F_{R\_R}$ is the vertical force of the right rear wheel $T_{F\_L}$ is the braking force of the left front wheel $T_{F\_R}$ is the braking force of the right front wheel$T_{R\_L}$ is the braking force of the left right wheel$T_{R\_R}$ is the braking force of the right rear wheel $S_{Brake}$ is the brake signal1: **while**
$S_{Brake}>0$
**do** 2: $Load\_1=F_{F\_L}/(F_{F\_L}+F_{F\_R}+F_{R\_L}+F_{R\_R})$; 3: $Load\_2=F_{F\_R}/(F_{F\_L}+F_{F\_R}+F_{R\_L}+F_{R\_R})$; 4: $Load\_3=F_{R\_L}/(F_{F\_L}+F_{F\_R}+F_{R\_L}+F_{R\_R})$; 5: $Load\_4=F_{R\_R}/(F_{F\_L}+F_{F\_R}+F_{R\_L}+F_{R\_R})$; 6: $T_{F\_L}=Load\_1*T_{Brake\_All}$; 7: $T_{F\_R}=Load\_2*T_{Brake\_All}$; 8: $T_{R\_L}=Load\_3*T_{Brake\_All}$; 9: $T_{R\_R}=Load\_4*T_{Brake\_All}$; 10: **end while** 11: $result=[T_{F\_L};T_{F\_R};T_{R\_L};T_{R\_R}]$; 12: **return**
result


## 4. Simulation and Experimentation

In this section, wheel slip rate is mainly used as the criterion for judging the merits of the vehicle braking force distribution strategy. In order to verify the rationality of the braking force distribution strategy proposed in this paper, the vehicle model of the four-wheeled truck is established by MATLAB/Simulink and TruckSim, and simulated and verified under FTP25 working conditions. In this paper, Changchun city road is used as the driving condition, and CANoe is used for the vehicle data acquisition, and a total of 56 data items, such as longitudinal vehicle speed and side/longitudinal speed of the mass on the spring are collected. The random forest algorithm with decision tree as 500 was used to filter the features among 56 data items, and a total of 26 data items (Longitudinal acceleration, vertical velocity of sprung mass and other parameters) with the greatest correlation with the four-wheel vertical force were filtered out to realize the dimensionality reduction of the data. By analyzing and comparing the simulation results of different parameter estimation methods and the braking distribution strategy of the vehicle, the GRNN strategy based on machine learning can achieve an accurate estimation of the nonlinear parameters of the vehicle, which provides a robust data basis for the braking force distribution and improves the safety and smoothness of the braking process of the vehicle. Please note that the simulations are performed on a computer equipped with an Intel i7-12700H processor and 16 GB of RAM.

### 4.1. Complete Vehicle Data Acquisition

**Figure 6:**
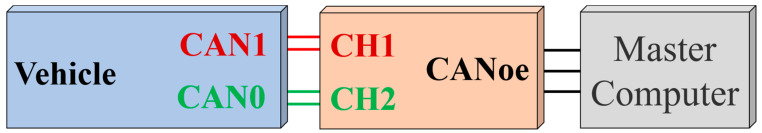
Structure diagram of the test program.(CAN0, CAN1, CH1 and CH2 are the interface of CAN).

**Figure 7:**
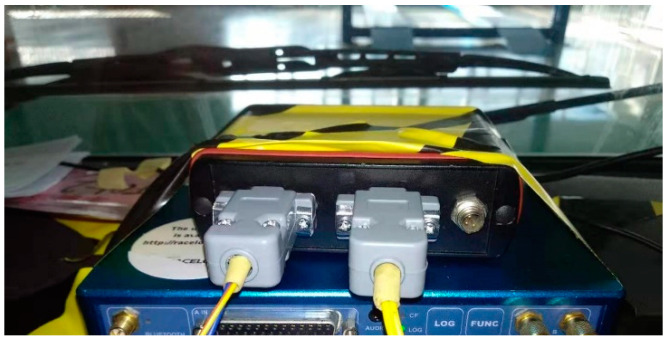
Physical diagram of the test hardware.

According to the requirements of the data set parameters and the preliminary analysis of the data quality, the CANoe tool is used to collect the data information of the vehicle. The test hardware scheme and the physical hardware are shown in Figure 6 and Figure 7, respectively.

**Figure 8:**
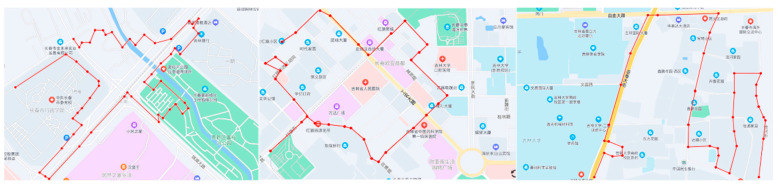
Roadmap of Changchun test conditions. (The red line is the actual route of the vehicle).

The experimental data collection of the vehicle is carried out with Changchun road conditions as the main driving conditions. The road map of Changchun conditions is shown in Figure 8. The collected information of individual parameters of the vehicle is saved and exported, and the interpolation and filtering processes are carried out to construct a reasonable data set.

### 4.2. Comparison of the Results of Estimation of Four-Wheel Vertical Force Parameters Based on GRNN

**Figure 9:**
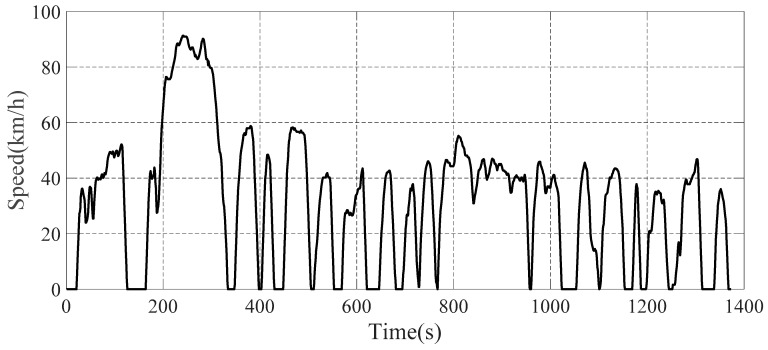
Simulated speed time course of the vehicle.

In the actual driving process, the vertical force situation of the four wheels directly affects the efficiency of the vehicle braking in order to give full play to the effect of the vehicle braking force and prevent the vehicle ABS from triggering leading to wheel locking, which affects the safety and smoothness of the vehicle. Among them, Longitudinal acceleration, vertical velocity of sprung mass and other parameters directly affect the force situation of the four wheels of the vehicle, and the force state of the four wheels presents complex nonlinear changes in the time dimension, which is difficult to be estimated accurately. This paper uses MATLAB/Simulink and TruckSim for joint simulation, and FTP72 is the simulation driving condition, as is shown in Figure 9.

**Figure 10:**
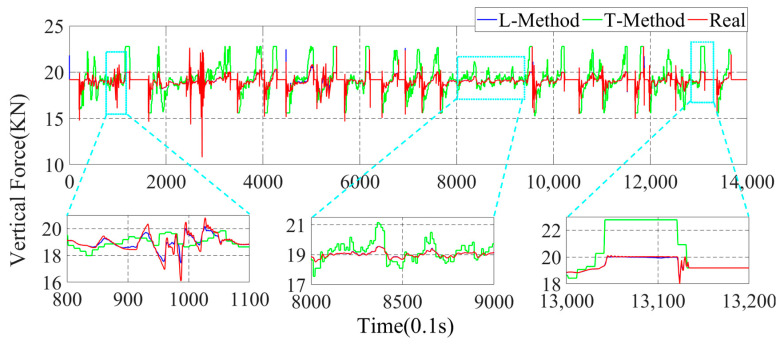
Parameter estimation of the left-front wheel vertical force of the vehicle.

**Figure 11:**
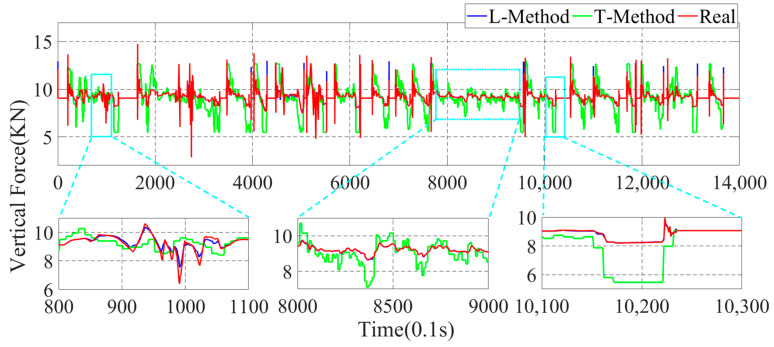
Parameter estimation of the left-rear wheel vertical force of the vehicle.

**Figure 12:**
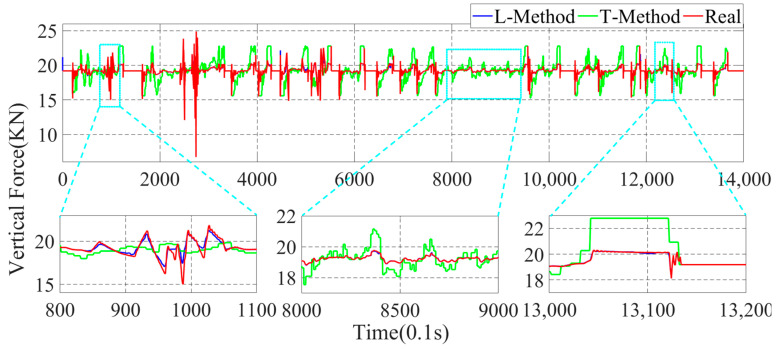
Parameter estimation of the vertical force of the right-front wheel of the vehicle.

**Figure 13:**
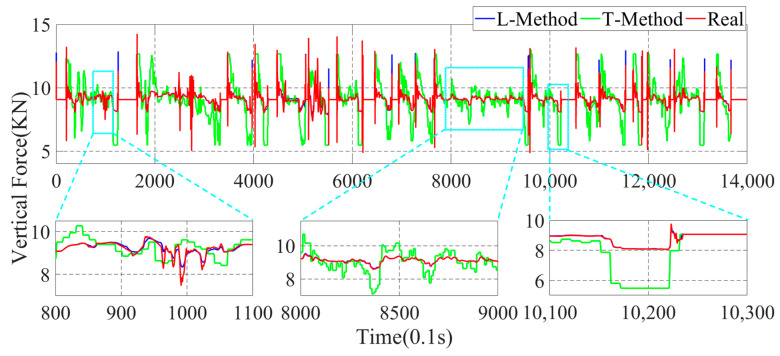
Parameter estimation of the right-rear wheel vertical force of the vehicle.

In this paper, the GRNN machine learning method (L-Method) is used for the estimation of the four-wheel force state parameters. To better illustrate the superiority of the data-driven method proposed in this paper, the more traditional moment Torque-balance method (T-Method) is used to estimate the four-wheel parameters. In the actual driving process of the vehicle, a time interval of 0.1 s is adopted to estimate once, and the parameter estimation results are shown in Figure 10, Figure 11, Figure 12 and Figure 13.

In Figure 10, Figure 11, Figure 12 and Figure 13, the parameter estimation of the four wheels presents different effects under different strategies. In Figure 10 and Figure 11, it can be seen that the overall vertical force of the left-front wheel is larger than that of the left-rear wheel during braking. In addition, the right-front and right-rear wheels in Figure 12 and Figure 13 exhibit the same characteristics. The reason for this is that the focus of the vehicle shifts forward during the braking process, resulting in the front wheels taking on more vertical force, yet the rear wheels have less vertical force. With the different braking intensities, the front and rear wheels’ vertical force changes out of different characteristics. From the above graph, it can be seen that in the 800.0 s–900.0 s interval, the variation of the vertical force of the four wheels is small. However, in the 1010.0 s–1030.0 s, it can be seen that the vertical force of the front and rear wheels exhibit a large variation due to the greater braking intensity.

In the above simulation results, the parameters estimation of the four-wheel vertical force of the vehicle based on the data-driven machine learning has perfect estimation accuracy, and it can be seen from Figure 11, Figure 12 and Figure 13 that the parameters estimation method based on the L-Method performs significantly during the driving process, and the trend of the estimated four-wheel vertical force is highly consistent with the actual four-wheel vertical force of the vehicle. Then, the T-Method has a relatively large error in the estimation of the four-wheel vertical force of the vehicle, and the estimation effect has low accuracy. In the 80.0 s–110.0 s interval, the four-wheel vertical force exhibits a certain amplitude of vibration due to the suspension of the vehicle. In the interval of 800.0 s–900.0 s, the four-wheel vertical forces of the vehicle have a small up-and-down amplitude, and the T-Method has the phenomenon of up-and-down vibration with large error. However, the L-Method shows a better estimation accuracy. In the 1010.0 s–1030.0 s, the L-Method also shows a high estimation accuracy.

Through the analysis of the above simulation results, the L-Method can accurately estimate the four-wheel vertical force values from the state information of the vehicle. Its change trend shows a high consistency with the real data. Accurately estimating the four-wheel vertical force of the vehicle provides a strong data base for the braking force distribution of the vehicle and improves control accuracy. 

### 4.3. Braking Force Distribution Results Comparison

**Figure 14:**
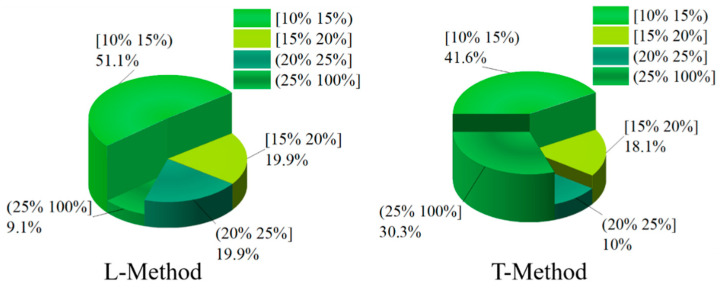
Ratio of front-left wheel slip under different control strategies.

**Figure 15:**
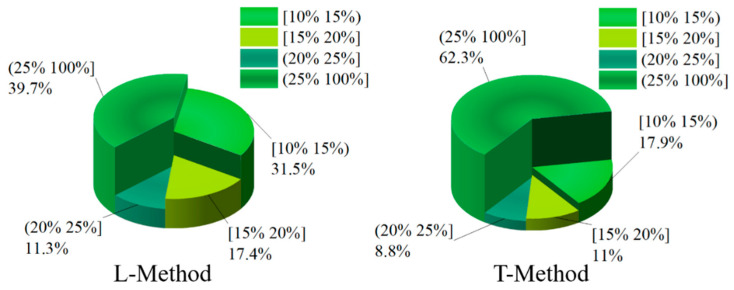
Ratio of left-rear wheel slip under different control strategies.

**Figure 16:**
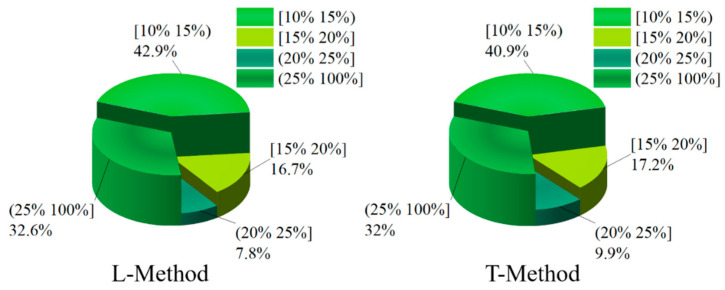
Ratio of right-front wheel slip under different control strategies.

**Figure 17:**
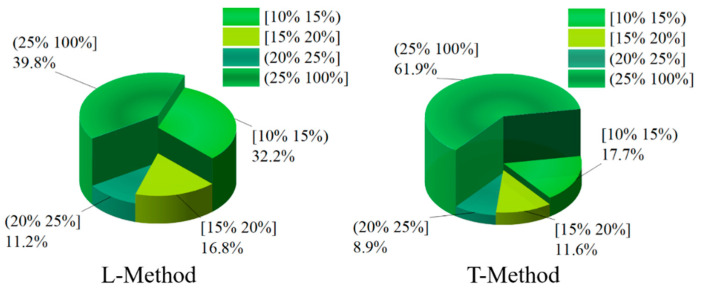
Ratio of right-rear wheel slip under different control strategies.

In order to deeply analyze the reasonableness of braking force distribution based on four-wheel state observation, give full play to the braking capacity of four-wheeled trucks and ensure the smoothness and safety of the vehicle. Slip rate is a direct manifestation of wheel clamping, and the braking force distribution strategy keeps the slip rate of four wheels of the truck within a reasonable range to prevent the wheels from reaching the clamping threshold, which affects the stability and safety of the vehicle. In this paper, the slip rate distribution is taken as the criterion for the superiority of the braking force distribution of the vehicle. In the braking process of the vehicle, in order to give full play to the braking efficiency of the vehicle, it is necessary to ensure that the slip rate of the four wheels is kept within a reasonable range. If the slip rate is too high, the ABS function will be triggered to keep the slip rate of the four wheels within a reasonable range of 15–20%. In order to further investigate the superiority of the proposed braking distribution control strategy of the vehicle, the slip rate of the four wheels of the vehicle is analyzed and compared. In this paper, the distribution of the slip rate of the vehicle is divided into four grade ranges, which are [10–15%), [15–20%], and (20–100%]. Among them, the slip rate is kept at [15–20%], and the vehicle can be fully braked with a better braking effect. In this paper, the slip rate distribution of four wheels is shown in Figure 14, Figure 15, Figure 16 and Figure 17 through the joint simulation of MATLAB/Simulink and TruckSim.

Figure 14 and Figure 15 show the distribution of the slip rate of the left-front wheel and the left-rear wheel under different control strategies. From Figure 14, the percentage of slip rate of the left-front wheel in the range of [15–20%] is 18.1% based on the T-Method. However, the percentage of slip rate of the left-front wheel in the range of [15–20%] is 19.9% based on the L-Method strategy, which has a better braking force effect. The slip rate in (25–100%] indicates that the wheel has obvious slippage, which seriously affects the braking effect of the vehicle, and the left-front wheel slip rate in the T-Method-based method occupies 30.3% in this interval. In contrast, based on the L-Method only occupies 9.1% in this interval, the braking force distribution strategy performs better. In addition, in the interval [20–25%], the percentage of left front wheel slip under the L-Method-based approach is still higher than that under the T-Method strategy.

As shown in Figure 15, the left-rear wheel still has a high percentage of 17.4% in the interval [15–20%] of the slip rate based on the L-Method. The left-rear wheel has 11% of the slip rate in the interval [15–20%] and 62.3% in the interval (25–100%), and the slip phenomenon of the left-rear wheel appears more frequently, and the braking effect is poor in the T-Method. In the interval (20–25%) of the slip rate, the proportion of the T-Method is higher than that of the L-Method, which is only 2.1% higher. On the whole, the braking force distribution strategy based on the L-Method shows a better control effect in the process of vehicle braking than that based on the T-Method.

Figure 16 and Figure 17 show the distribution of the slip rate of the right-front wheel and the right-rear wheel under different control strategies. From Figure 16, the slip rate of the right-front wheel under the T-Method is 17.2% in the interval of [15–20%]. However, the percentage of the L-Method is 16.7% in the interval of [15–20%]. In the interval of (25–100%), the both control strategies show similar control effects, and the percentage of the L-Method and T-Method are 32.6% and 32%, respectively. In the [10–15%) and (20–25%] slip rate intervals, the right-front wheel under both control strategies has similar percentage cases. As can be seen from Figure 17, the right-rear wheel shares of the L-Method is significantly higher than that of the T-Method in the [15–20%] slip rate interval, with the shares of 16.6% and 11.6%, respectively. In both the [10–15%) and (20–25%] slip rate intervals, the right-rear wheel shares under the L-Method is significantly higher than that under the T-Method.

Through the analysis and comparison of the above results, the braking force distribution strategy based on the L-Method proposed in this paper has a better effect, which can be more distributed in the interval of [15–20%] and less distributed in the interval of [25–100%] for the four wheels of the vehicle, which can effectively avoid the phenomenon of the vehicle slipping several times during the braking process and can give full play to the braking potential of the vehicle.

## 5. Conclusions

In this paper, a braking force distribution strategy is proposed for a four-wheel truck to fully utilize the braking potential. In order to better realize the accurate estimation of the high nonlinear parameters in the vehicle, a machine learning method is used for the accurate estimation of the four-wheel vertical force parameters of the vehicle to provide accurate data for the braking force distribution of the four wheels. The method in this paper provides a new method for the current research of vehicle parameter estimation. In order to verify that the proposed method can have the correct estimation of the four-wheel pendant force values of the vehicle and the reasonable distribution of braking force, a joint simulation was conducted using MATLAB/Simulink and TruckSim, and through the comparison and analysis of the simulation results, the L-Method was able to keep the wheel slip rate within a reasonable range and give full play to the braking potential of the vehicle.

In the study of this paper, the method is suitable for the same type of trucks, the application range is narrow. In the future research, the new method will be explored for more models. Meanwhile, in the process of braking force distribution, the accuracy of vehicle state parameter estimation directly affects the control effect. The estimation accuracy of complex nonlinear parameters of the vehicle will be further improved to provide more accurate parameters of the vehicle for the control of the braking force, and further develop the braking potential of the vehicle.

## Figures and Tables

**Table 1:** Vehicle parameter information.

Parameter	Unit	Value
Vehicle Curb weight	kg	4455
Vehicle Maximum velocity	km/h	140
Wheel rolling radius	m	0.51
Frontal area	m^2^	6.8
Engine maximum power	kW	175
Engine maximum torque	Nm	740.6
First gear ratio	-	3.74
Second gear ratio	-	2
Third gear ratio	-	1.34
Fourth gear ratio	-	1
Fifth gear ratio	-	0.77
Sixth gear ratio	-	0.63
Reverse gear ratio	-	3.54
Final drive ratio	-	5.571

**Table 2:** Parameter values.

Parameter	Value
g	9.8
f	0.02
δ	1.3
$C_{D}$	0.5
A	6.8

**Table 3:** ABS operating characteristics.

Brake Pressure	Pressurization Valve	Pressure Reducing Valve
Pressurization	Off	Off
Pressure retention	On	Off
Decompression	On	On

**Table 4:** Brake related parameter setting.

Parameter	$A_{b}$	$\eta_{b}$	$\mu_{b}$	$r_{b}$	$c_{b}$
Value	0.005	0.99	0.25	0.3	3
Unit	$m^{2}$	-	-	m	-

## Data Availability

Not applicable.
